# Determining appropriate timing of adaptive radiation therapy for nasopharyngeal carcinoma during intensity-modulated radiation therapy

**DOI:** 10.1186/s13014-015-0498-1

**Published:** 2015-09-17

**Authors:** Huixian Huang, Heming Lu, Guosheng Feng, Hailan Jiang, Jiaxin Chen, Jinjian Cheng, Qiang Pang, Zhiping Lu, Junzhao Gu, Luxing Peng, Shan Deng, Ying Mo, Danling Wu, Yinglin Wei

**Affiliations:** Departments of Radiation Oncology, People’s Hospital of Guangxi Zhuang Autonomous Region, Nanning, China; Department of Medical Oncology, People’s Hospital of Guangxi Zhuang Autonomous Region, Nanning, China

## Abstract

**Background:**

To determine appropriate timing of an adaptive radiation therapy (ART) replan by evaluating anatomic and dosimetric changes of target volumes and organs at risk (OARs) during intensity-modulated radiation therapy (IMRT) for nasopharyngeal carcinoma (NPC).

**Methods:**

Nineteen NPC patients were recruited. Each patient had repeat computed tomography (CT) scans after each five fractions and at treatment completion. Automatic re-contouring the targets and OARs by using deformable registration algorithm was conducted through CT-CT fusion. Anatomic changes were assessed by comparing the initial CT and repeated CT. Hybrid plans with re-contouring were generated and the dose-volume histograms (DVH) of the hybrid plan and the original plan were compared.

**Results:**

Progressive volume reductions in gross target volume for primary disease (GTVnx), gross target volume for involved lymph nodes (GTVnd), and parotids were observed over time. Comparing with the original plan, each hybrid plan had no significant difference in homogeneity index (HI) for all the targets. Some parameters for planning target volumes for primary disease and high-risk clinical target volume (PTVnx and PTV1, respectively) improved significantly, notably starting from the 10th fraction. These parameters included mean dose (Dmean), dose to 95 % of the volume (D95), percentage of the volume receiving 95 % of the prescription dose (V95), and conformity index (CI) for PTVnx, and Dmean, D95, and CI for PTV1. The dosimetric parameters for PTVnd remained the same in general except for D95 and V95 which had significant improvement at specific time points; whereas for PTV2, similar trend of dosimetric changes was also observed. Dose to some OARs increased significantly at some time points.

**Conclusions:**

There were significant anatomic and dosimetric changes in the targets and OARs. The target dose coverage in the hybrid plans did not get worse, but overdose occurred in some critical structures. Significant dosimetric changes should be considered as a trigger point at which ART replanning is indicated. D95/V95/CI for PTV2, Dmax for the brain stem, spinal cord, right eyeball and left lens, and Dmean/V30 for the parotids and glottis were taken into account for predicting the need for ART. Two replans at the 5th and 15th fractions were suggested.

## Background

Radiation therapy (RT) is considered a main treatment approach in the management of nasopharyngeal carcinoma (NPC). Intensity-modulated radiation therapy (IMRT), formally introduced in clinical practice since early 1990s, has now replaced conventional radiation therapy (CRT) and three-dimensional radiation therapy (3D-CRT) and become a standard treatment for NPC. This technique provides adequate target coverage while maintaining steep dose gradients at the border between the target and adjacent normal tissues, through which dose escalation for the targets may be achieved without delivering excessive dose to the organs at risk (OARs) [1]. Numerous studies have demonstrated that IMRT results in a decreased incidence of radiation-induced side effects and an improvement in quality of life (QOL) for NPC patients, comparing with CRT or 3D-CRT [2–6].

However, NPC patients may experience significant anatomical changes throughout the entire treatment course. These changes include the shrinkage of the primary disease and metastatic lymph nodes, external contour because of significant weight loss, and displacement/size of the normal structures. As a consequence of the anatomic changes, significant difference between the actual delivered dose and the initial planned dose would be anticipated, which may ultimately result in underdose to the targets and/or overdose to the critical structures. Wang et al. [7] compared a repeated CT after treatment at a dose of 40 Gy with the initial planning CT for 20 patients with locally advanced NPC, and found that the dose coverage of the targets remained unchanged; whereas the dose delivered to the parotid glands and spinal cord increased significantly. Excessive irradiation to OARs increased the risk of incidence of radiation-induced complications such as xerostomia and myelitis, which may have a great negative impact on QOL [8–12].

It is suggested that the initial planning based on pretreatment condition may not truly reflect the dosimetric variations during the course of IMRT. Thus adaptive radiation therapy (ART), a plan modification and implementation according to tumor response and anatomic changes of normal structures, becomes particularly important. In recent years, many studies have focused on ART for NPC patients, however, the optimal timing and frequency of ART remains unanswered [13–19]. In this prospective study, we aimed to determine appropriate timing to perform an ART replan by evaluating anatomic and dosimetric changes of the target volumes and OARs during IMRT for NPC.

## Methods

### Eligibility criteria

Patients with histologically proven NPC and treated with curative IMRT were enrolled into this prospective study. Inclusion criteria were as follows: age ≥18 years; Eastern Cooperative Oncology Group (ECOG) Performance Status 0–2; stages I-IVb according to the 2010 AJCC Staging System. Patients diagnosed with, or treated for other malignances, or treated with non-IMRT techniques were excluded in the study. Written informed consent was obtained for all patients. The study was approved by the Institutional Review Board (IRB) .

### Immobilization and simulation

All patients were immobilized in a supine position with the head in a neutral position with a tailored thermoplastic mask covering the head, neck, and shoulders. Intravenous contrast-enhanced CT using 2 mm slice from the vertex to the manubriosternal joint was performed for planning. The CT data were imported to the a treatment planning system (Pinnacle^3^, version 9.2).

### Delineation of the targets and OARs

The target delineation was in accordance with the International Commission on Radiation Units and Measurements Reports 50 and 62. Briefly, the primary gross volume (GTVnx) and the involved cervical lymphadenopathy (GTVnd) included all known gross disease as determined by the imaging, clinical, and endoscopic findings. The high-risk clinical target volume (CTV1) was defined as GTVnx plus 5-mm margin and entire nasopharyngeal mucosa plus 5-mm submucosal volume. The low-risk clinical target volume (CTV2) covered CTV1, entire nasopharynx, parapharyngeal space, pterygopalatine fossa, posterior third of the nasal cavity and maxillary sinuses, inferior sphenoid sinus, posterior ethmoid sinus, skull base, and anterior half of the clivus. CTV2 also covered elective neck nodal regions, including bilateral retropharyngeal lymph nodes and ipsilateral levels II, III, and Va for node-negative neck, or full length of ipsilateral neck for node-positive neck. Level Ib was not routinely irradiated unless there was confirmed lymphadenopathy in level Ib, or large metastatic node size (≥3 cm)/extracapsular spread was present in level IIa. PTVnx, PTVnd, PTV1, and PTV2 were generated by adding 5-mm margin to GTVnx, GTVnd, CTV1, and CTV2, respectively. Care was taken to ensure at least 5-mm gap was present between the PTVs and the skin. The contoured critical structures included the brain stem, chiasm, optic nerves, spinal cord, eyes, lens, parotid glands, oral cavity, larynx, mandible, and temporomandibular joints.

### Treatment design and delivery

The plans were designed and optimized using the Pinnacle inverse planning system. The prescribed radiation dose was 69.76 Gy at 2.18 Gy per fraction delivered to the PTVnx and PTVnd, and 60.8 Gy at 1.9 Gy per fraction delivered to the PTV1. The PTV2 was treated to 54.4 Gy at 1.7 Gy per fractions. All patients were treated once daily, five fractions weekly. Dose constrains to the critical structures were within the tolerance according to the RTOG 0225 protocol, and efforts were made to meet the criteria as closely as possible. IMRT was delivered via seven fixed-gantry angles with an Elekta Synergy Linear Accelerator (Elekta Ltd.) with step-and-shoot treatment techniques.

### Acquiring and processing CT data during IMRT

Repeat CT scans were acquired for each patient with the same mask and isocenter as the initial simulation CT scan after each five fractions and at the end of the full treatment course using a 24-slice CT scanner (Somatom Sensation Open, Siemens Medical Solutions, Erlangen, Germany). The datasets were denoted as CT-1, CT-2, CT-3, CT-4, CT-5, and CT-6, respectively. Each new CT dataset was registered with the initial planning CT dataset through VoxAlign Deformation Engine provided by the MiM Maestro software. Auto-propagating the planning contours on the new CT was conducted and manual modification was performed if needed. Changes in the volume of GTVnx, GTVnd, and parotid and shift of the parotid centroid were calculated by comparing the new CT and the planning CT.

A hybrid IMRT plan was generated by superimposing the initial treatment plan (Plan-0) to each repeated new CT image. After recalculation of the dose distribution on the new CT images, the dose delivered by the hybrid plans to the redelineated target volumes and OARs were recorded and analyzed (adaptive replanning was not suitable for CT-6 since the CT-6 images were acquired at the end of IMRT. There were only 5 hybrid plans, namely Plan-1, Plan-2, Plan-3, Plan-4, and Plan-5).

### Statistical analysis

The Kolmogorov-Smirnov test was used to test data for normality. Mean ± standard deviation was used for data with normal distribution; whereas median (interquartile range) was used for data with skewed distribution. A paired sample *T*-test or Wilcoxon rank-sum test was chosen based on the data types. The correlation between weight loss and parameters associated with anatomic changes was estimated using Pearson’s correlation. Analysis of variance (ANOVA) was performed to determine significant dosimetric changes at any time point (trigger point) of the entire treatment course. A probability value less than 0.05 was considered significant. Analyses were performed using SPSS 19.0 software (SPSS, Inc., Chicago, IL, USA).

## Results

### Patient characteristics

Between August 2012 and December 2013, a total of 19 patients diagnosed with undifferentiated non-keratinizing NPC were enrolled into this study. There were 11 men and 8 women with median age of 46 years (range, 22–70). Stage distributions according to the 2010 AJCC Staging System were as follows: stages II, 3 patients; stage III, 13 patients; and stage IV, 3 patients. Concurrent platinum-based chemotherapy was given to 17 patients, and no chemotherapy to 2 patients. The characteristics of the patient cohort are listed in Table 1.

**Table 1:** Patient characteristics

Characteristics	No. of patients	Percent
Sex		
Male	11	57.9
Femal	8	42.1
Age (years)		
Range	22–70	
Median	46	
KPS score		
100	0	0
90	13	68.4
80	4	21.1
70	2	10.5
T stage		
T1	2	10.5
T2	10	52.6
T3	6	31.6
T4	1	5.3
N stage		
N0	1	5.3
N1	2	10.5
N2	14	73.7
N3	2	10.5
AJCC stage group		
I	0	0
II	3	15.8
III	13	68.4
IVA	1	5.3
IVB	2	10.5
Concurrent chemo		
None	2	10.5
Platinum-based chemo	17	89.5

### Changes in volume of GTVnx and GTVnd

Steady volume reduction in GTVnx and GTVnd was observed over time. Compared with the baseline, the volumes of GTVnx and GTVnd at the end of IMRT were decreased significantly by 65.6 ± 13.3 % and 72.7 ± 13.3 % (*p* = 0.001 and 0.046), corresponding to a reduction rate of 17.3 ± 10.4 %/week and 22.2 ± 15.0 %/week, respectively.

### Changes in volume and displacement of the parotid

At the treatment completion, the volume of the left parotid decreased by 38.0 ± 15.3 %, corresponding to a reduction rate of 7.9 ± 9.6 %/week; whereas for the right parotid, the volume reduction was 39.2 ± 14.7 %, corresponding to a reduction rate of 7.8 ± 11.3 %/week. The most significant volume reduction occurred at the third week, with its value of 15.8 ± 6.8 % (*p* = 0.016) and 14.4 ± 7.6 % (*p* = 0.003) for the left and right parotids, respectively.

**Fig. 1:**
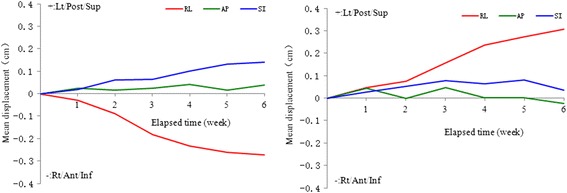
Centroid displacements of the parotid glands. Centroid displacements of the left (left part) and right parotid gland (right part) in the RL, AP, and SI directions throughout the entire treatment course. Lt=left, Post = posterior, Sup = superior, Rt = right, Ant = anterior, Inf = inferior

The displacements of the centroid for the left parotid were −0.27 ± 0.25 cm, 0.04 ± 0.12 cm, and 0.14 ± 0.20 cm in the right-left (RL), anterior-posterior (AP), and superior-inferior (SI) directions, respectively. Significant shifts were noticed in the RL and AP directions (*p* = 0.001 and 0.018). While for the right parotid, the displacements of its centroid were 0.31 ± 0.19 cm, 0.01 ± 0.21 cm, and 0.03 ± 0.20 cm in the RL, AP, and SI directions, respectively. Significant shift occurred in the AP direction (*p* < 0.001). The centroid displacement for the parotids is illustrated in Fig. 1.

### Weight loss during the treatment course

Gradual weight loss was observed during the course of IMRT, with an average loss of 1.5 ± 2.2 %/week. Compared with the baseline, the percentages of weight loss after the 1st, 2nd, 3rd, 4th, and 5th weeks and at the completion of treatment were 1.7 ± 1.5 % (*p* < 0.001), 2.5 ± 1.9 % (*p* < 0.001), 3.9 ± 2.2 % (*p* < 0.001), 4.8 ± 2.8 % (*p* < 0.001), 6.2 ± 3.3 % (*p* < 0.001), and 8.3 ± 5.1 % (*p* < 0.001), respectively.

Weight loss correlated significantly with reductions in the volume of parotids and their centroid shift towards the medial direction. No correlation was observed between the weight loss and the volumetric changes of GTVnx and GTVnd (Table 2).

**Table 2:** Correlation between weight loss and anatomic changes of the parotids and tumor

	Anatomic change	R^2*^	*P* value
	Volume reduction in PG-Lt	0.359	<0.001
	Volume reduction in PG-Rt	0.294	<0.001
Weight loss	Shift towards medial direction for PG-Lt centroid	0.325	<0.001
	Shift towards medial direction for PG-Rt centroid	0.594	<0.001
	Volume reduction in GTVnx	0.006	0.467
	Volume reduction in GTVnd	0.0004	0.842

^*^Pearson correlation coefficient

*PG-Lt* left parotid gland, *PG-Rt* right parotid gland

### Dosimetric changes in the target volumes

There was no significant difference in each homogeneity index (HI) for all the targets between the original plan and the hybrid plan. Some parameters for PTVnx and PTV1 improved significantly as treatment continued, notably starting from the 10th fraction. These parameters included mean dose (Dmean), dose to 95 % of the volume (D95), percentage of the volume receiving 95 % of the prescription dose (V95), and conformity index (CI) for PTVnx, and Dmean, D95, and CI for PTV1. Other parameters including percentage of the volume receiving >110 % of the prescription dose (V110) for PTVnx, and V95 and V110 for PTV1 changed less or not significantly. The dosimetric parameters for PTVnd remained the same in general except for D95 which had significant improvement at the 15th and 25th fraction, and for V95 which had significant improvement starting from the 10th fraction to 25th fraction; whereas for PTV2, a similar trend of dosimetric changes was also observed (Table 3).

**Table 3:** Dosimetric changes in different target volumes

	Plan-0	Plan-1	Plan-2	Plan-3	Plan-4	Plan-5
PTVnx						
Dmean (Gy)	71.17 ± 1.49	71.31 ± 1.44	71.41 ± 1.43	71.50 ± 1.40	71.60 ± 1.42	71.61 ± 1.44
*P* value		0.073	0.004	0.001	<0.001	0.001
D95 (Gy)	67.40 ± 2.48	67.54 ± 2.32	67.64 ± 2.25	67.86 ± 2.26	68.23 ± 2.04	68.18 ± 2.17
*P* value		0.404	0.256	0.040	0.004	0.015
V110 (%)	0.29 ± 0.65	0.30 ± 0.64	0.34 ± 0.75	0.29 ± 0.70	0.31 ± 0.86	0.32 ± 0.88
*P* value		0.447	0.102	0.990	0.887	0.852
V95 (%)	95.97 ± 5.06	96.29 ± 4.76	96.68 ± 4.4	97.21 ± 3.77	97.72 ± 3.59	97.48 ± 4.34
*P* value		0.158	0.032	0.013	0.005	0.027
HI	1.09 ± 0.03	1.09 ± 0.03	1.09 ± 0.03	1.09 ± 0.03	1.09 ± 0.03	1.09 ± 0.03
*P* value		0.244	0.244	0.116	0.258	0.678
CI	0.76 ± 0.24	0.77 ± 0.24	0.78 ± 0.23	0.80 ± 0.22	0.81 ± 0.22	0.82 ± 0.21
*P* value		0.141	0.044	0.005	0.004	0.004
PTVnd						
Dmean (Gy)	71.60 ± .08	71.51 ± 1.08	71.56 ± 1.12	71.68 ± 1.08	71.73 ± 1.25	71.81 ± 1.22
*P* value		0.134	0.634	0.334	0.312	0.090
D95 (Gy)	66.43 ± 3.16	66.88 ± 3.31	66.89 ± 3.27	67.03 ± 3.27	67.27 ± 3.98	67.60 ± 3.57
*P* value		0.093	0.116	0.025	0.061	0.011
V110 (%)	0.74 ± 1.43	0.70 ± 1.29	0.71 ± 1.37	0.74 ± 1.45	0.8 ± 1.66	0.76 ± 1.65
*P* value		0.528	0.386	0.984	0.984	0.902
V95 (%)	95.09 ± 4.56	96.02 ± 4.21	96.02 ± 4.43	96.09 ± 4.47	96.66 ± 5.44	96.75 ± 5.85
*P* value		0.066	0.034	0.050	0.010	0.033
HI	1.10 ± 0.04	1.10 ± 0.04	1.10 ± 0.04	1.10 ± 0.04	1.10 ± 0.03	1.10 ± 0.03
*P* value		0.282	0.159	0.056	0.186	0.150
CI	0.80 ± 0.20	0.79 ± 0.18	0.80 ± 0.18	0.81 ± 0.18	0.82 ± 0.19	0.83 ± 0.18
*P* value		0.355	0.951	0.244	0.063	0.102
PTV1						
Dmean (Gy)	68.06 ± 2.22	68.38 ± 2.03	68.52 ± 2.04	68.62 ± 1.96	68.69 ± 2.05	68.74 ± 2.06
*P* value		0.014	0.001	0.003	0.001	0.001
D95 (Gy)	60.00 ± 4.12	60.63 ± 3.91	61.04 ± 4.11	61.08 ± 4.33	61.24 ± 4.41	61.20 ± 4.43
*P* value		0.146	0.020	0.004	0.017	0.009
V95 (%)	95.58 ± 4.23	96.80 ± 2.97	96.90 ± 3.17	96.69 ± 3.73	96.79 ± 3.59	96.93 ± 3.46
*P* value		0.093	0.071	0.051	0.077	0.019
HI	1.25 ± 0.03	1.25 ± 0.03	1.25 ± 0.03	1.25 ± 0.03	1.25 ± 0.03	1.25 ± 0.03
*P* value		0.478	0.706	0.258	0.053	0.086
CI	0.90 ± 0.07	0.92 ± 0.05	0.93 ± 0.06	0.93 ± 0.05	0.93 ± 0.06	0.93 ± 0.06
*P* value		0.040	0.038	0.006	0.046	0.008
PTV2						
Dmean (Gy)	60.21 ± 6.65	59.98 ± 6.73	60.07 ± 6.90	60.10 ± 6.80	60.08 ± 6.94	60.20 ± 6.64
*P* value		0.071	0.345	0.412	0.379	0.940
D95 (Gy)	47.47 ± 6.42	46.73 ± 6.12	46.94 ± 6.18	46.89 ± 6.21	46.75 ± 16.1	46.61 ± 6.06
*P* value		0.008	0.069	0.038	0.020	0.023
V95 (%)	92.48 ± 0.97	91.39 ± 1.10	91.74 ± 1.12	91.84 ± 0.92	91.56 ± 1.25	91.52 ± 0.52
*P* value		0.015	0.071	0.088	0.028	0.057
HI	1.42 ± 0.05	1.42 ± 0.05	1.42 ± 0.05	1.42 ± 0.05	1.42 ± 0.05	1.42 ± 0.05
*P* value		0.334	0.229	0.167	0.173	0.130
CI	0.87 ± 0.13	0.86 ± 0.13	0.86 ± 0.13	0.86 ± 0.13	0.86 ± 0.13	0.86 ± 0.13
*P* value		0.032	0.184	0.271	0.082	0.173

Difference was calculated by comparing Plan-0 and each hybrid plan

*Dmean* mean dose, *D95* dose to 95 % of the volume, *V110* percentage of the volume receiving more than 110 % of the prescription dose, *V95* percentage of the volume receiving 95 % of the prescription dose, *HI* homogeneity index, *CI* conformity index

### Dosimetric changes in OARs

Dmean for the brain stem and spinal cord increased significantly over the IMRT course, occurring as early as at the 15th and 5th fractions, respectively. Dmean and V30 for the left and right parotids increased significantly, beginning at the fifteenth and twentieth fraction, respectively. For the glottis, right eye, and left lens, significant increase in Dmax or Dmean was found in some time point (s) during the treatment. Table 4 illustrates the dosimetric changes in some OARs. No significant differences were found in dosimetric parameters between the initial treatment plan and the hybrid plan in other OARs including the temporal lobes, optic nerves, optic chiasm, left eye, right lens, cochleae, mandible, temporomandibular joints, esophagus, oral cavity, supraglottis, and subglottis (not shown in Table 4).

**Table 4:** Dosimetric changes in OARs

OARs	Parameter	Plan-0	Plan-1	Plan-2	Plan-3	Plan-4	Plan-5
BT	Dmax (Gy)	53.21 ± 3.51	53.48 ± 4.59	53.51 ± 3.87	55.19 ± 3.40	55.84 ± 3.12	56.54 ± 3.72
	*P* value		0.613	0.590	<0.001	<0.001	<0.001
SC	Dmax (Gy)	40.74 ± 3.22	41.34 ± 3.36	43.08 ± 6.27	42.84 ± 5.72	42.66 ± 4.88	42.24 ± 3.87
	*P* value		0.017	0.041	0.030	0.012	0.001
PG-Lt	Dmean (Gy)	34.31 ± 5.53	34.33 ± 5.58	35.23 ± 6.07	36.72 ± 6.67	37.17 ± 7.47	37.40 ± 7.62
	*P* value		0.990	0.421	0.031	0.027	0.020
	V30 (%)	47.08 ± 2.06	47.57 ± 2.95	50.35 ± 5.00	52.38 ± 3.93	51.33 ± 4.05	53.37 ± 4.72
	*P* value		0.773	0.229	0.017	0.063	0.010
PG-Rt	Dmean (Gy)	32.98 ± 3.58	33.31 ± 4.88	33.44 ± 4.61	34.69 ± 5.02	35.40 ± 3.93	38.58 ± 1.07
	*P* value		0.673	0.533	0.083	0.003	0.038
	V30 (%)	43.72 ± 6.49	44.79 ± 9.94	45.19 ± 9.16	48.34 ± 0.86	50.10 ± 8.51	51.61 ± 0.05
	*P* value		0.499	0.298	0.051	0.002	0.001
Glottis	Dmean (Gy)	40.97 ± 7.27	41.42 ± 7.67	41.51 ± 7.60	41.72 ± 7.46	41.45 ± 7.46	42.61 ± 9.69
	*P* value		0.167	0.062	0.035	0.100	0.137
Eye-Rt	Dmax (Gy)	27.55 ± 8.34	30.30 ± 8.33	28.48 ± 8.30	30.20 ± 8.88	30.14 ± 9.45	29.79 ± 0.25
	*P* value		0.057	0.321	0.014	0.016	0.090
Lens-Lt	Dmax (Gy)	6.87 ± 1.50	8.35 ± 3.40	8.04 ± 3.01	8.41 ± 3.27	8.56 ± 2.83	8.60 ± 3.91
	*P* value		0.046	0.035	0.041	0.008	0.082

*OARs* organs at risk, *BT* brain stem, *SC* spinal cord, *PG-Lt* left parotid gland, *PG-Rt* right parotid gland, *Eye-Rt* right eye, *Lens-Lt* left lens

### Timing of adaptive treatment replanning

It is reasonable to postulate that any anatomic changes during the treatment course may not certainly result in marked dosimetric changes. So ART may not be necessary for a patient who has no significant dosimetric changes at some time point even if statistical anatomic changes exist at the same time. Based on this concept, we considered a trigger point at which significant dosimetric variation for a specific parameter was present as an indicator for ART replanning. First of all, dosimetric parameters in Plan-0 were compared with those in the following hybrid plans separately until statistical differences were found. For example, if significant difference was found in the target dose coverage or overdose to the OARs between Plan-0 and Plan-1, adaptive replanning would be initiated after the 5th fraction (here the 5th fraction was considered a trigger point). Then parameters in Plan-1 were compared with those in the following hybrid plans until another trigger point was found, and so on. In this way, the trigger points for each selected parameter throughout the treatment course could be identified. Table 5 illustrates the suggested trigger points at which adaptive replanning would be designed. However, for practical consideration, two replans at the 5th and 15th fractions were proposed. This was largely because most of the trigger points occurred at these time points.

## Discussion

Similar to other head-and-neck cancers, patients with NPC disease also experience marked changes during IMRT. These changes include shrinkage of the primary disease and involved lymph nodes, weight loss, and geometric/volumetric variations of the OARs. Fung et al. [14] acquired daily megavoltage CT (MVCT) images and registered them to the corresponding planning CT images for 30 NPC patients treated with helical tomotherapy. At the end of treatment, there was a volume reduction of 35.70 ± 20.06 % for the posterolateral wall of nasopharynx (P-NP) which was a surrogate for the primary disease, corresponding to a daily reduction of 0.99 ± 0.55 %. In a study by Cheng et al. [20], the mean shrinkages of the nasopharyngeal disease and metastatic lymph nodes were 9.1 and 16.2 % at 30 Gy, and 13.1 and 28.7 % at 50 Gy, respectively. Barker et al. [19] found that the GTV for head-and-neck cancer treated with definitive external beam RT decreased throughout the course of fractionated RT, at a median rate of 0.2 cm^3^. On the last day of treatment, the median volume loss of the initial GTV was 69.5 % (range, 9.9–91.9 %). This was consistent with our findings. In the present study, progressive tumor regression for GTVnx and GTVnd was observed over time. The volumes of GTVnx and GTVnd at the end of IMRT were decreased significantly by 65.6 ± 13.3 % and 72.7 ± 13.3 % (*p* = 0.001 and 0.046), corresponding to reduction rates of 17.3 ± 10.4 %/week and 22.2 ± 15.0 %/week, respectively.

Weight loss is a common event for head-and-neck cancer patients when RT is given with or without concurrent chemotherapy. Ng et al. [21] found that at the end of RT, 82 % of NPC patients had significant weight loss and were in negative energy balance, which persisted for more than 6 months. Although NPC patients who received IMRT lost less weight than those who received conventional RT, weight loss in patients treated with IMRT should not be underestimated. In a study by Qiu et al. [22], weight loss of 5.81 ± 2.34 kg was observed. Cheng et al. [20] found that most NPC patients who were treated with IMRT experienced significant weight loss during the RT course. Compared with the baseline, mean weight loss at 30 and 50 Gy were 5.4 and 9.3 %, respectively. In the present study, we found an average weight loss of 1.5 ± 2.2 % per week. Besides, there was a significant weight loss at each time point from the first week to the end of treatment course, indicating that the patients experienced progressive weight loss. Patients in our study lost less body weight than those in Cheng’s study. This may be attributed to the discrepancy in the patients’ baseline physical status, severity of complications caused by treatment itself, and support care between the two studies. Significant weight loss during RT may have an negative impact on treatment outcome, including lower quality-of-life (QOL) scores, poorer treatment compliance, prolonged recovery time and hospital stay, and poorer prognosis [23–26].

The parotids are the most frequently changed organs in NPC patients throughout the treatment course, both in the volume and in the displacement. Wang et al. [7] showed a volume reduction of 14.7 % for the left parotid and 18.2 % for the right parotid during IMRT for locally advanced NPC by comparing the initial planning CT and a repeat CT obtained after treatment at a dose of 40 Gy. Lu et al. [16] found a volume reduction of 35.1 ± 20.0 % and 24.6 ± 11.9 % for the left and right parotids, respectively, after 25 fractions. Similar reduction trends for the parotid could also be reflected by another study in which the mean percentage volume loss was 47.54 ± 14.27 % at the treatment completion, and the mean loss rate was estimated to be 1.35 ± 0.39 %/day. In addition, the mean center of mass (COM) of the parotid shifted progressively towards the medial and superior aspects during treatment. Also the mean medial and superior displacement for both sides of the parotid were 0.34 ± 0.27 cm and 0.24 ± 0.39 cm, respectively (both P < 0.001) [14]. In our study, the volume of both sides of the parotid at the treatment completion decreased significantly, comparing with the baseline. We found that the most significant volume reduction occurred at the 3rd week. Apart from volumetric variation, the displacement of the centroid for the parotids also changed. Significant shifts were found in the AP direction for the right parotid, and in the RL and AP directions for the left parotid.

It has been reported that shrinkage of the parotid and shift of its centroid are correlated with weight loss. Barker et al. [19] found that in head-and-neck cancer, the medial shift in parotid COM correlated highly with weight loss during fractionated RT course. Similar findings were also shown in our study. Weight loss significantly correlated with the shrinkage of parotids and their centroid shift towards the medial direction, but no correlation was observed between the weight loss and the volumetric changes of GTVnx and GTVnd. The parotids may be exposed to a high dose region due to their medial shift or volume reduction during the IMRT course. Significant weight loss may be used as a surrogate for a large medial parotid shift or larger parotid volume loss, implying that modified replanning may be needed to avoid excessive irradiation to the parotid.

Significant anatomic changes during the treatment course in NPC patients may affect the dose distribution. The initial plan based on pretreatment CT images that are only snapshots of the patient’s anatomy at a static time point do not precisely reflect the actual dose distribution during fractionated IMRT. Fung et al. [13] created two new adaptive plans (PII-ART and PIII-ART) for 10 NPC patients undergoing Hi-Art Tomotherapy based on up-to-date CT images and contours and used these plans for treatment in phase two (PII; after 25th fraction) and phase three (PIII; after 35th fraction), respectively. Two hybrid plans (PII-NART and PIII-NART) were generated using the original contours pasted on the PII- and PIII-CT sets by CT-CT fusion. Dosimetric comparisons were made between the NART plans and the corresponding ART plans. They found that without replanning, the doses to D95 for all the target volumes were increased with better dose uniformity, whereas the OARs received higher doses compared with the corresponding ART plans. The total dose to D1 for the brain stem and spinal cord significantly increased by 7.87 ± 7.26 and 10.69 ± 6.72 %, respectively. There were also significantly increased maximum doses to the optic chiasm and pituitary gland, and significantly increased mean doses to both sides of the parotid. Cheng et al. [20] performed CT and MRI scans at 30 Gy and 50 Gy intervals for 19 NPC patients treated with IMRT. When comparing the initial plan with the hybrid plans which were generated by superimposing the initial plan to the repeat CT images, they found that the hybrid plans demonstrated significantly higher dose to most of target volumes with greater dose inhomogeneity, higher maximum doses to the spinal cord and brainstem, and higher median doses to the parotid. Wang et al. [7] noticed that after IMRT at the 40 Gy dose point, dose coverage of all the targets remained unchanged, whereas the dose delivered to the parotid glands and spinal cord increased significantly. The results were in line with ours in this study. Findings mentioned above indicated that although the actually delivered doses to the targets may be higher or at least not worse than the planned ones, doses to OARs, particularly the parotids, spinal cord, and brain stem, however, significantly increased.

Determining an appropriate time point at which ART is intervened in a timely manner is critical to ensure that the planned dose to the targets and OARs can be delivered faithfully throughout the entire IMRT course. Workflow for ART includes re-simulating, re-contouring, and treatment re-designing, which is a time-consuming and heavy workload process. Thus daily ART is not always applicable in many cancer centers. Many reports in recent years suggested that mid-to-late phase of the treatment course was appropriate timing for ART. Lu et al. [16] recommended that ART should be initiated after 25 fractions of IMRT to ensure adaptive doses to the targets and critical normal tissues. Cheng et al. [20] showed that doses to some OARs such as the brain stem, spinal cord and parotids were significantly increased at 30 Gy during the course of IMRT, while the target coverage remained adequate. So replanning was suggested at this time point. In another study by Fung et al., significant anatomic changes were found at the 9th, 19th, and 29th fractions. The authors thus recommended ART replanning at these three time points. Contrary to Fung’s study, we selected parameters only related with dose distributions as the endpoint to determine whether a replan was needed. As explained in the previous section, significant anatomic changes may not certainly result in remarkable changes in dosimetric effects. So only dosimetric changes were chosen as determinant for ART replanning in this study. Bearing this concept in mind, we identified the trigger points at which ART replanning would be initiated. However, for practical consideration, two replans at the 5th and 15th fractions were proposed. This was largely because most of the trigger points occurred at these time points, as shown in Table 5.

**Table 5:** Suggested trigger points for adaptive replanning

Parameter	Week 1	Week 2	Week 3	Week 4	Week 5
PTV2 (D95/V95/CI)	*				
Brain stem (Dmax)			*		*
Spinal cord (Dmax)	*				
PG-Lt (Dmean/V30)			*		
PG-Rt (Dmean/V30)				*	
Glottis (Dmean)			*		
Eye-Rt (Dmax)			*		
Lens-Lt (Dmax)	*				
Sum	3	0	4	1	1

Note that parameters without statistically significant difference analyzed by ANOVA were not shown in the table

^*^Significant difference at a trigger point, indicating necessity of replanning

It should be noted that all patients in the present study were treated with the original plan. The repeat CT scans during IMRT were only used for research, and no ART replanning was prepared for patient treatment. Whether ART replanning can transfer into dosimetric or clinical benefits remains unknown. To answer these questions, a further study is underway to investigate the impact of ART on dosimetric parameters and clinical outcomes.

## Conclusions

In summary, there were significant anatomic and dosimetric changes in the targets and OARs during the course of IMRT. Weight loss significantly correlated with the shrinkage of parotids and their centroid shift towards the medial direction. The target dose coverage in the hybrid plans did not get worse, comparing with the original plan, but overdose occurred in some critical structures. Significant dosimetric changes should be considered as a trigger point at which ART replanning would be initiated. Based on the dosimetric analysis, D95/V95/CI for PTV2, Dmax for the brain stem, spinal cord, right eyeball and left lens, and Dmean/V30 for the parotids and glottis were taken into account for predicting the need for ART. Two replans at the 5th and 15th fractions were suggested for performing ART.
